# Supplementary material for: Decomposing a San Francisco estuary microbiome using long-read metagenomics reveals species- and strain-level dominance from picoeukaryotes to viruses
Source: mSystems. 2024 Aug 19;9(9):e00242-24. doi: 10.1128/msystems.00242-24 (PMC11406994; doi:10.1128/msystems.00242-24)
Supplement: Supplemental material — Figures S1-S10 and supplemental methods. [file msystems.00242-24-s0001.pdf]

Supplementary Files for “Decomposing a San Francisco Estuary microbiome using long read metagenomics reveals species- and strain-level dominance from picoeukaryotes to viruses”

Lauren M. Lui*†[1] and Torben N. Nielsen*†[1]

[1]Environmental Genomics and Systems Biology Division, Lawrence Berkeley National Laboratory, Berkeley, CA, USA.

†Contributed equally to the manuscript

*Correspondence: lmlui@lbl.gov, torben@lbl.gov

Supplementary Figure 1: Electropherogram of Femto Pulse separation of size-selected DNA from USGS Station 36.

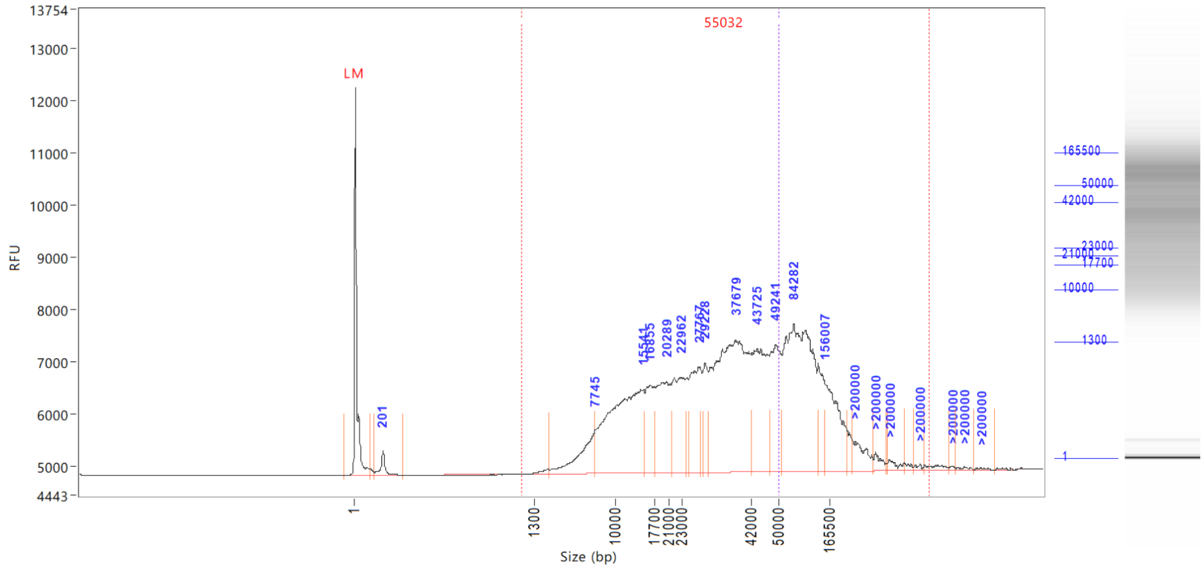

Supplementary Figure 2: Electropherogram of Femto Pulse separation of Nanopore library made with SQK-LSK112 kit.

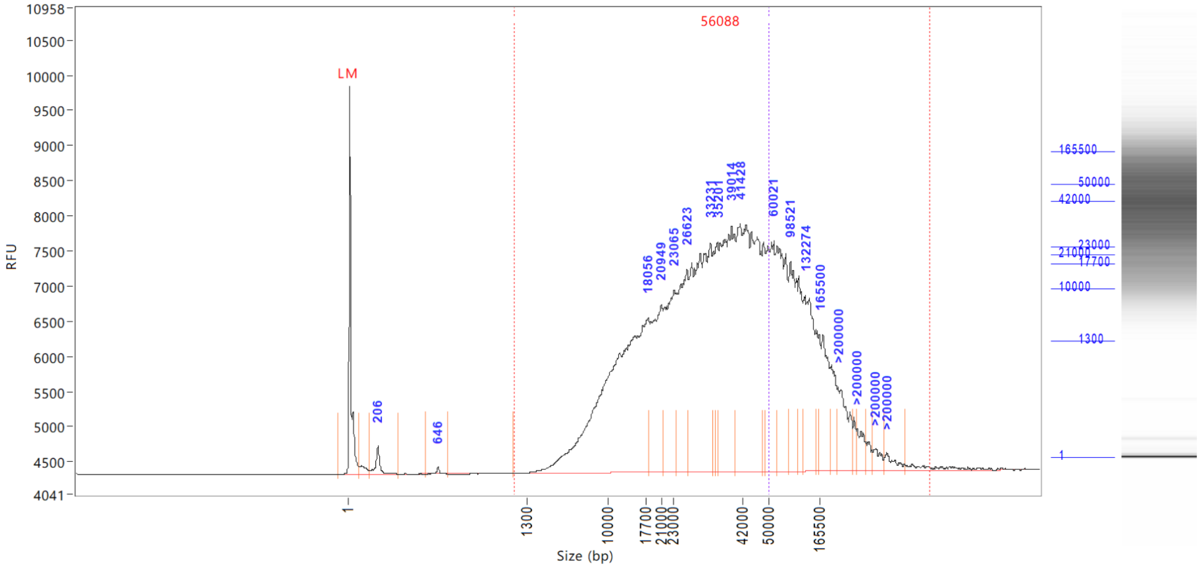

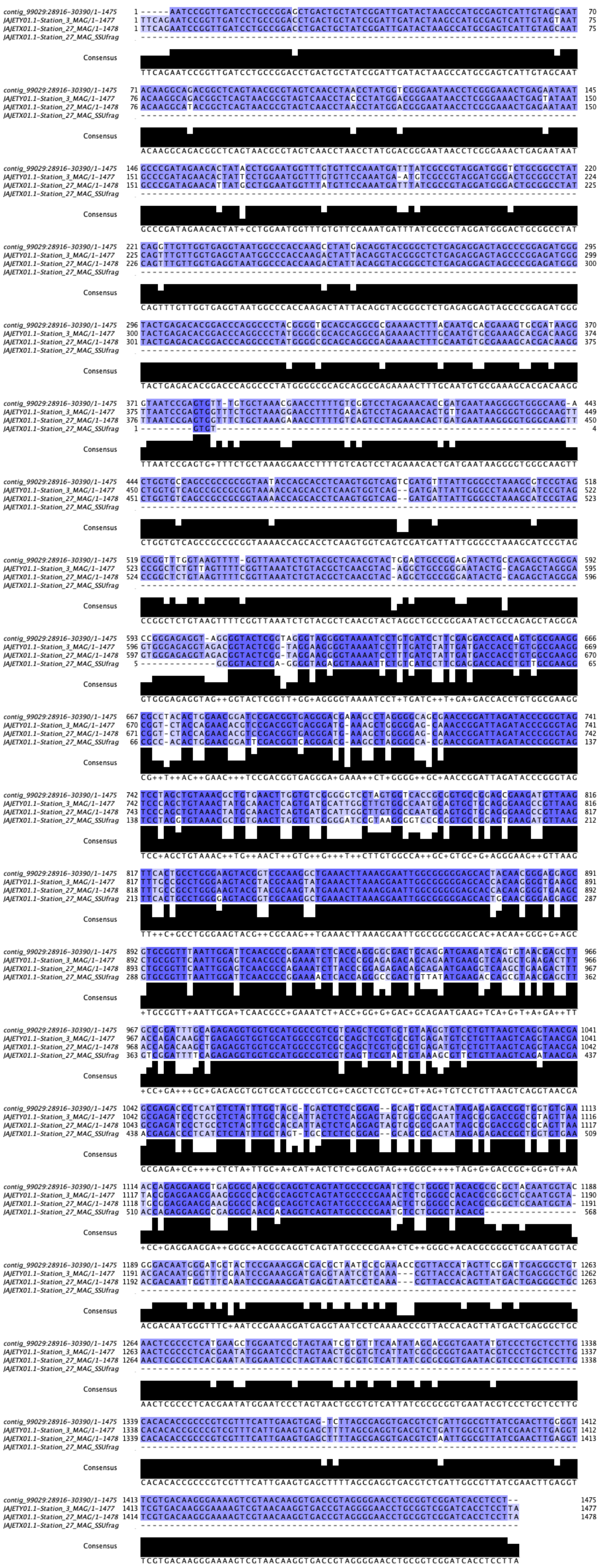

Supplementary Figure 3: Alignment of *Nitrosopumilus* SSU with SSUs from Rasmussen et al. 2022. Rasmussen et al. 2021 Microbial Ecology reported on dominant archaeal OTU (Thaumarchaeota) from the South Bay of the San Francisco Estuary. The authors subsequently reanalyzed the 16S data via amplicon sequence variant (ASV) analysis in a paper focused on two ammonia-oxidizing archaea MAGs[1]. The ASV analysis indicated that of the Thaumarchaeota ASVs, the most abundant ranked 8th of all ASVs (ASV8), while the next most abundant Thaumarchaeota ASV ranked 754. This suggests that this ASV is similar to the dominant OTU. We wanted to see if the SSU in our dataset matched either the ASV8 or OTU. Since the OTU and ASV sequences were not published by Rasmussen et al, we aligned our SSU with SSUs from two ammonia-oxidizing archaea MAGs that Rasmussen et al assembled from USGS Station 3 and 27 water samples. The Station 3 MAG had one full length SSU, while the Station 27 MAG had one full length SSU and one 568 bp fragment. Rasmussen et al. report that the Station 27 MAG exactly matches ASV8 from their 16S study, which we assume is to the full length SSU, as the fragment does not cover the entirety of the V4-V5 region. The SSU from our dataset had only ~86% identity to both full length SSUs and ~91% identity to the fragment, so it is likely a different genus or species[2]. We note that the taxonomy output both by SILVA and MMseqs2 are not consistent, as the *Nitrosopumilus* contigs are classified under the Crenarchaeota and Thermoproteota, respectively, for the phylum, rather than Nitrososphaerota or Thaumarchaeota that is used by the International Committee on Systematics of Prokaryotes[3].

Supplementary Figure 4: Eukaryotic phyla of contigs classified by Kaiju and number of contigs detected for each species. Taxonomic class is listed on the x-axis and species with >100 contigs are labeled with the genus.

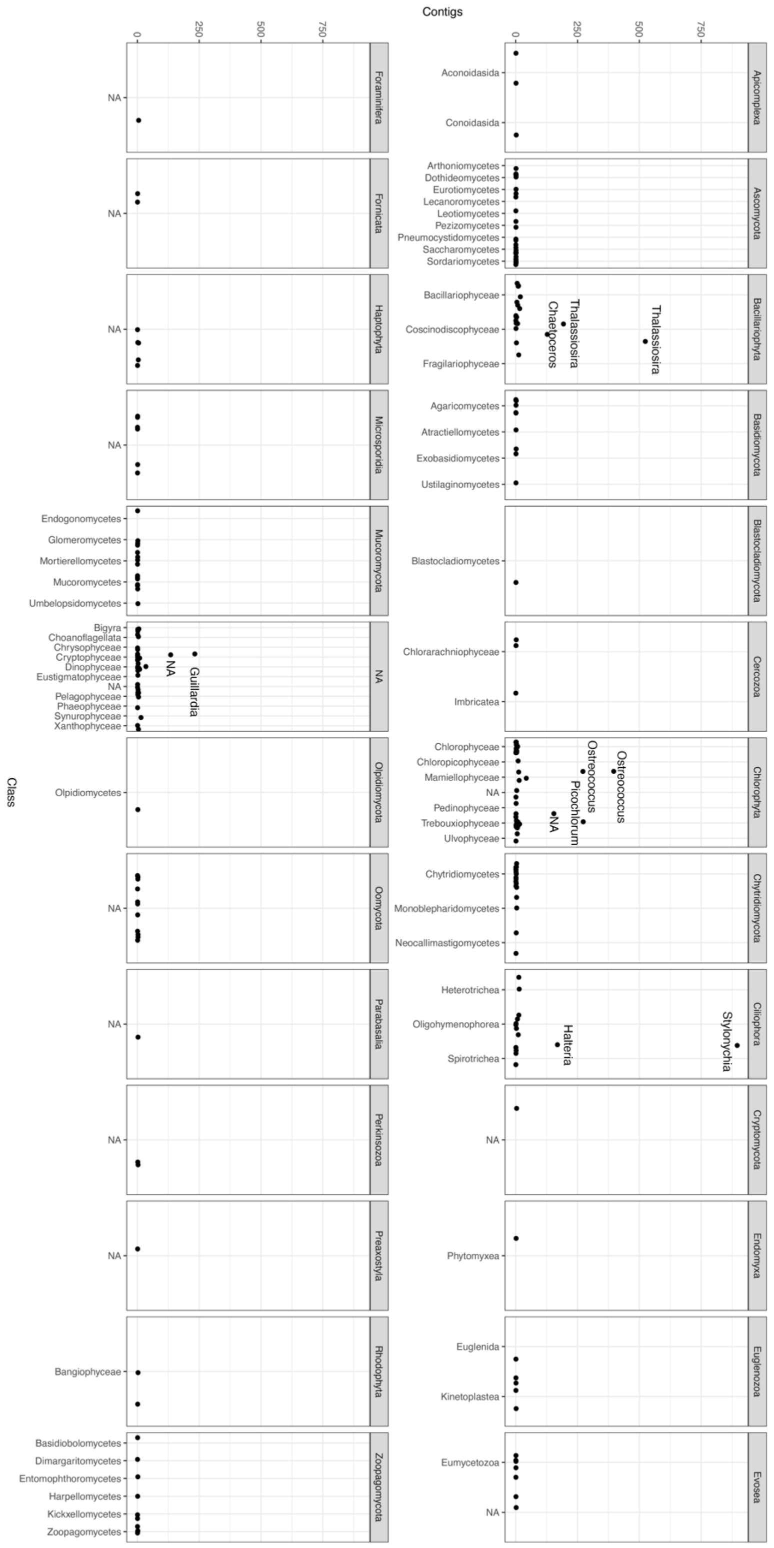

Supplementary Figure 5: Coverage vs Length (bp) of Giant Viruses in Station 36 metagenome. All contigs classified as viral by geNomad longer than 300 Kbp are shown. Circular contigs are represented by black circles.

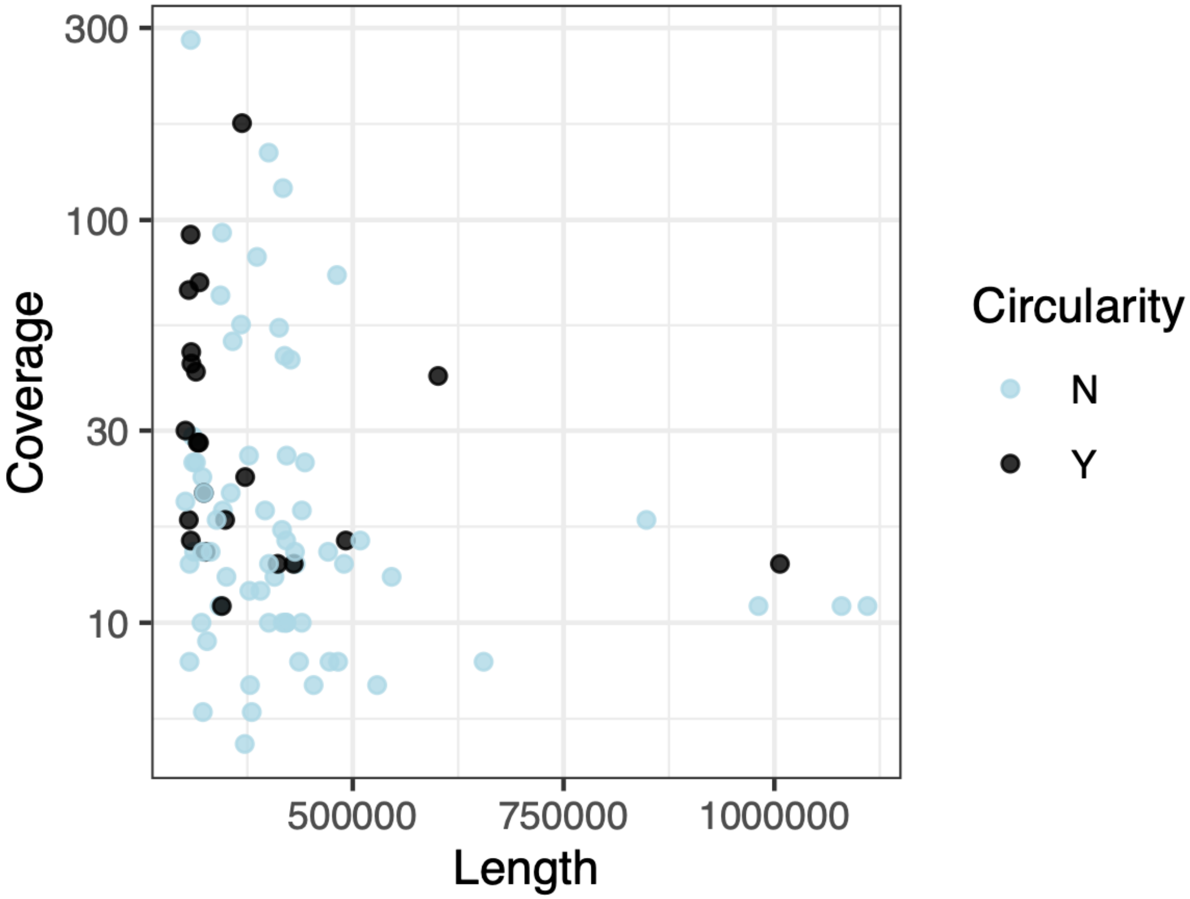

Supplementary Figure 6: Viral Taxonomy of the Station 36 metagenome. (A) Viral Realms detected. (B) Breakdown of Realm *Duplodnaviria.* (C) Breakdown of Realm *Monodnaviria*. (D) Breakdown of Realm *Varidnaviria*.

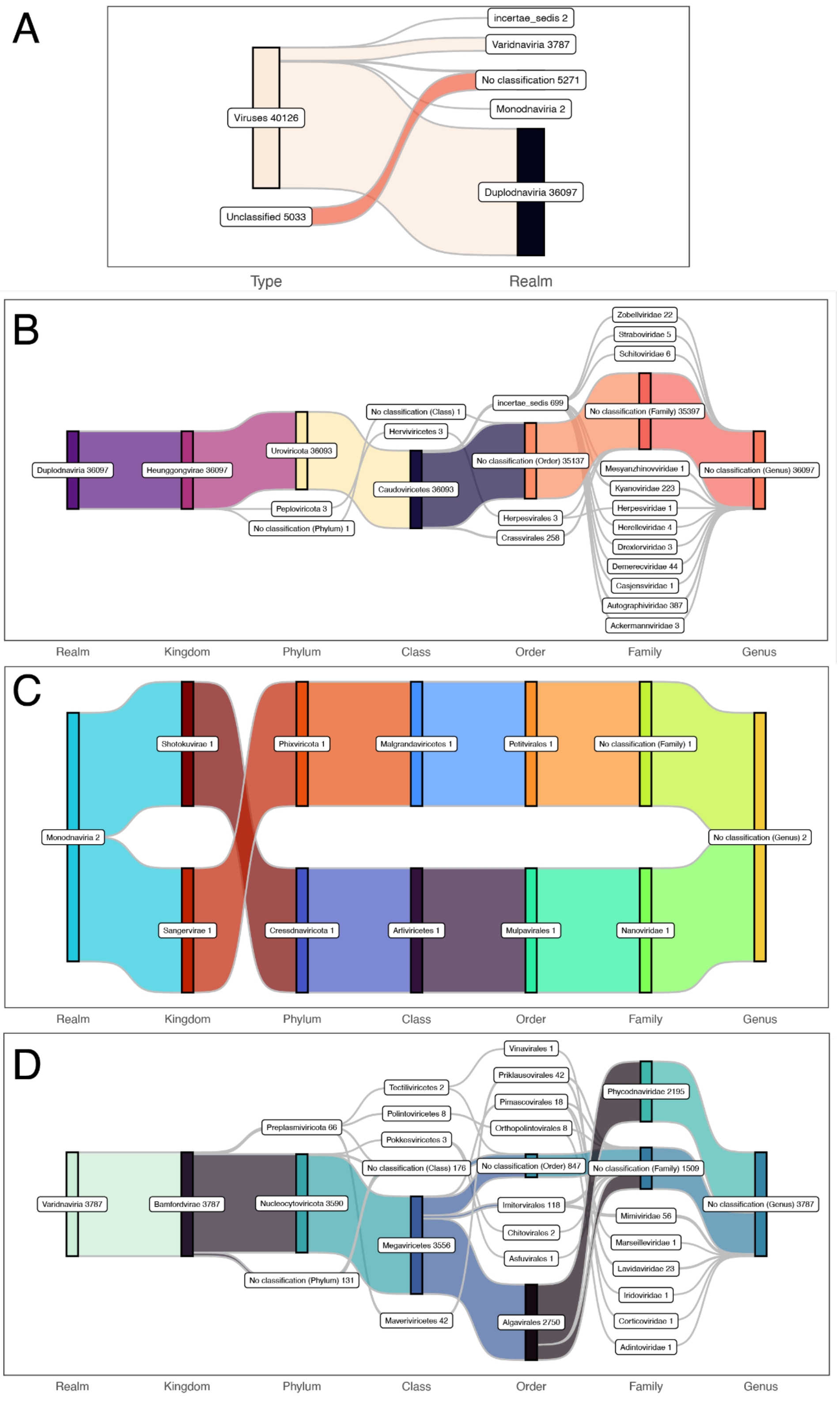

Supplementary Figure 7: *Phycodnaviridae* populations plotted by contig coverage and length (bp).

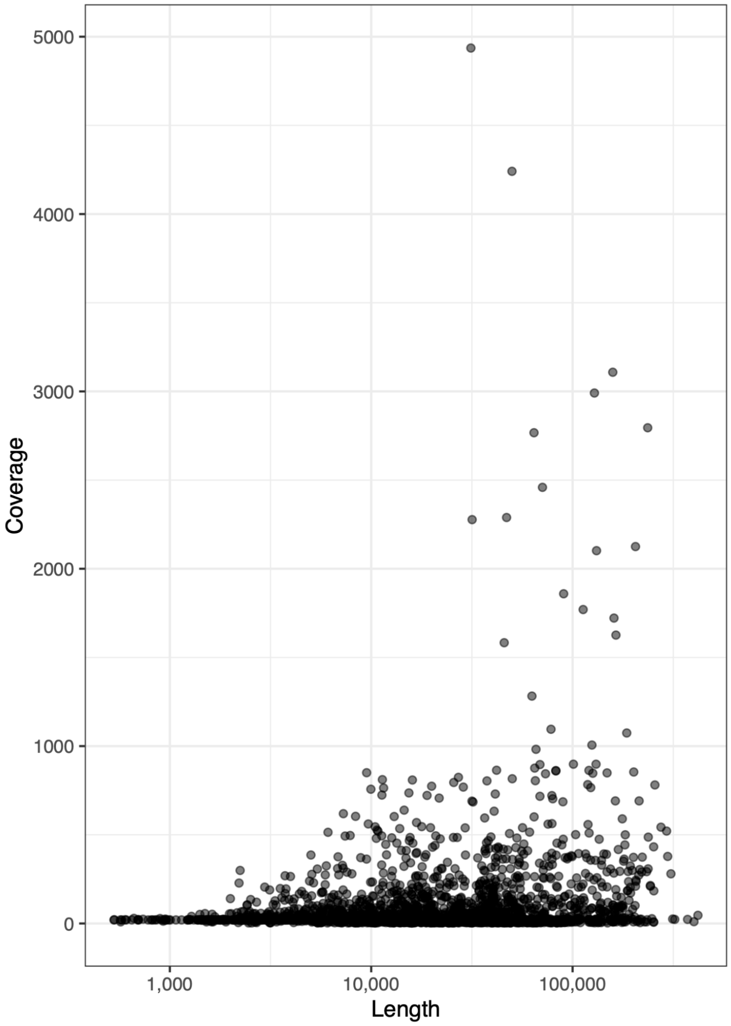

Supplementary Figure 8: *Proteobacteria* phage populations plotted by coverage and taxonomic family classification. This plot is provided as an example of the dominance of specific phage strains, rather than all strains in a taxonomic group being dominant at the same time.

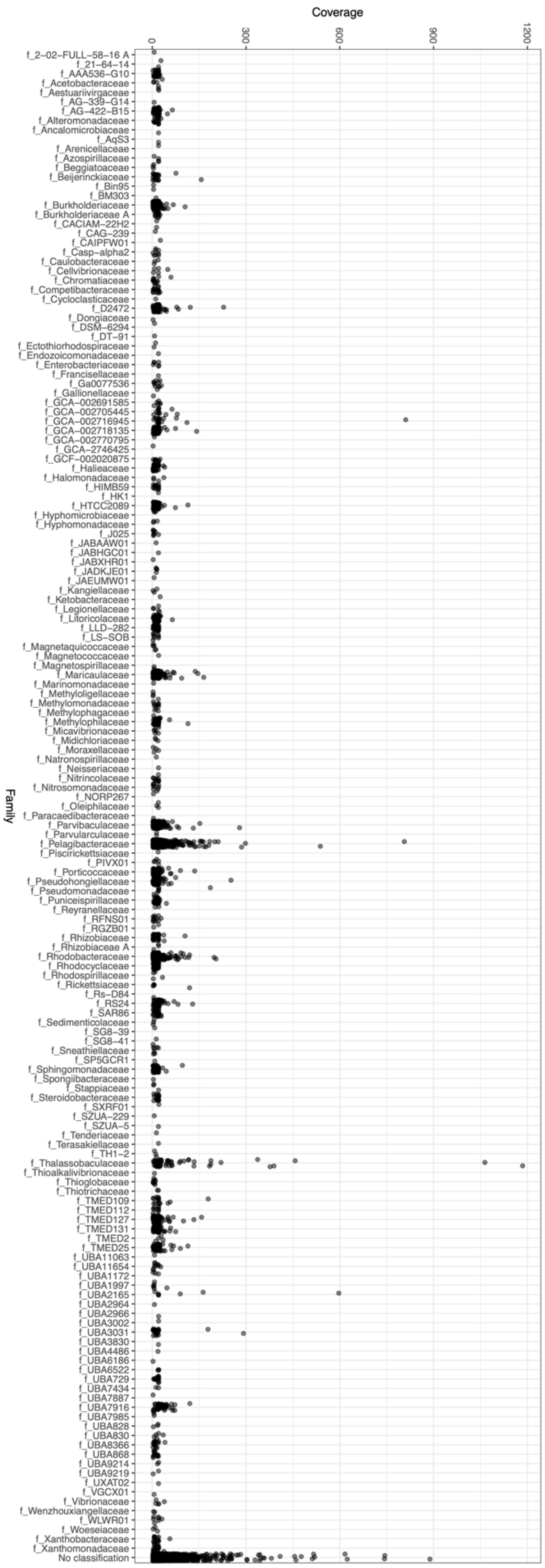

Supplementary Methods

Taxonomic classification of contigs at the Superkingdom level

As described in the main text, to classify the taxonomy of contigs we used geNomad, mmSeqs2, Kaiju, and manual curation based on SSUs and BLAST hits. In total, the assembly consisted of 107,977 contigs. First, we used geNomad, a deep neural network method, to classify contigs as mobile genetic elements (*e.g.*, plasmids and viruses) and to assign viral taxonomy. The authors of geNomad did extensive curation of their training data from genomes, plasmids, and viruses. Second, we used MMseqs2, which assigns taxonomy based on protein genes. GTDB was used as the reference database, so this classification was biased towards bacteria and archaea. Third, we used Kaiju to classify eukaryotic contigs. We used the Kaiju database that contains a subset of the NCBI BLAST nr database containing all proteins from archaea, bacteria, viruses, fungal, and microeukaryotic reference genomes (db_nr_euk). Theoretically, this method should be the best at assigning eukaryotic classifications to the contigs. Finally, we manually curated contigs as either mitochondria or chloroplasts by using NCBI BLAST[4] on their SSU genes.

1. geNomad results:
    - 1963 Plasmids
    - 46476 Viruses
    - Note: Viral taxonomy is discussed in the main text
2. MMseqs2 results (using GTDB as the database):
    - 85445 Bacteria
    - 1646 Archaea
    - 6493 Root
    - 1698 No rank
    - 12695 Unclassified by mmseqs2
3. Kaiju results (using nr_euk as the database):
    - 44322 Bacteria
    - 751 Archaea
    - 5822 Eukaryota
    - 23499 Viruses
    - 8176 Cannot be assigned to a non-viral species
    - 25407 Unclassified
4. Manual curation of SSU containing contigs:
    - 493 Bacteria
    - 1 Archaea
    - 69 Eukaryota
    - 23 Chloroplasts
    - 59 Mitochondria

- Note: This list only contains contigs with full length SSUs to ensure accuracy. These numbers also take into account contigs that have more than one SSU

We had a number of assumptions about the classifiers that influenced how we interpreted the results:

1. **geNomad was the best at classifying contigs as viral or plasmid given the careful curation of plasmid and viral sequences going into the training data.** This assumption is also because (1) we did not focus on using Kaiju to classify viruses and did not use their viral specific database when running Kaiju on our contigs, and (2) Kaiju accuracy was not benchmarked on classification of viral sequences. Most Kaiju benchmarks were on sensitivity and accuracy of prokaryotic phylogeny (see Menzel et al 2016. Nat. Communications). We also assume that some of the MMseqs2 contigs were classified as bacterial or archaeal because often viruses pick up genes from their hosts.
2. **MMseqs2 is the best at classifying contigs as bacterial or archaeal, except if the contigs is viral, and Kaiju will be better at classifying contigs as eukaryotic, because of the databases used**. Given that we used the GTDB database and MMseqs2 algorithm relies on finding specific proteins in a contig and determining if there is a close match in the database, we consider this algorithm more accurate at prokaryotic classification than Kaiju, at least if a close genome exists in the GTDB database and the contig is long enough to contain enough identifying genes. For bacterial and archaeal genomes, the GTDB database is more highly curated than the Kaiju database we used (contains sequences from NCBI BLAST nr database of bacteria and archaea), and thus is likely more accurate for those taxonomies. Since we used the Kaiju database that includes eukaryotic sequences, it will automatically be more accurate at classifying contigs as eukaryotic than the MMseqs2 results.
3. **In all cases, we assume that sequences that were only classified at the superkingdom level (*e.g.,* a sequence classified as Bacteria with no phylum or lower taxonomic rank assigned) are ambiguous.** Taxonomy of sequences which do not have a close genome in the database (such as the genus or family level), will depend on the last common ancestor (LCA) algorithm used, and these are different between Kaiju and MMseqs2. We do not take a stance on which of these algorithms are more accurate.

First, we started with the contigs that we manually curated based on their SSUs, along with SILVA and BLAST taxonomy assignments. Since we manually curated these, we consider these accurate. We consider chloroplasts and mitochondria as eukaryotic.

<u>Manual curation</u>

- 493 Bacteria
- 1 Archaea
- 151 Eukaryota
- 107332 Unclassified

Next we added in the geNomad results for the viruses and plasmids. Eight contigs that were assigned as a plasmid and one that was assigned as a virus had SSUs, so these contigs were excluded from the counts of viral sequences.

We started with geNomad after the manual curation because many of the sequences classified as viral by geNomad were classified as archaeal or bacterial by MMseqs2, likely because some of these sequences also have some archaeal and bacterial genes. It is possible that some of the geNomad classification is not accurate, but we do not have an easy way to evaluate the accuracy, short of searching every contig for hallmark viral genes and manual curation. Of the 46,475 contigs that were classified as viral by geNomad, 3,697 were unclassified, 18,734 were classified at the superkingdom level, 4,231 as root, and 441 had no rank, so 27,103 (58%) of these sequences we would already consider ambiguous already by MMseqs2 assignments. So this number would be a more conservative accounting for the number of viruses, and we keep this in mind going forwards with the analysis.

There were 1,963 contigs that were classified as plasmids by geNomad. Of these, 369 contigs (18.8%) had an ambiguous classification. We carry these forward as ambiguous prokaryotic contigs. Three of the contigs were classified at the archaeal species level and 1,591 were classified as bacteria at the phylum level or below.

<u>Manual curation + geNomad results</u>

- 2084 Bacteria
- 4 Archaea
- 151 Eukaryota
- 369 Unassigned prokaryotic sequences (plasmids)
- 46475 Viruses (or 27103, conservatively)
- 58894 Unclassified

Next, we added in the MMseqs2 assignments for Bacteria and Archaea. In total, MMseqs2 assigned taxonomy to 95,282 contigs, so 12,695 contigs were not classifiable by MMseqs2. Removing the contigs that were already accounted for by SSU analysis and geNomad, 49,990 contigs were assigned taxonomy. 10,822 contigs were assigned as no rank, root, or at the superkingdom level so we considered these ambiguous. That left 38,902 contigs assigned as bacterial and 312 as archaeal.

<u>Manual curation + geNomad + MMseqs2</u>

- 40986 Bacteria
- 316 Archaea
- 151 Eukaryota
- 369 Unassigned prokaryotic sequences (plasmids)
- 46475 Viruses (or 27103, conservatively)
- 19680 Unclassified

Finally, we added in the Kaiju results for eukaryotic contigs. A total of 5,822 contigs were assigned eukaryotic by Kaju. Cross-referencing the specific contig assignments for eukaryotes required matching the NCBI taxonomic IDs in the raw output assigned to eukaryotic taxonomy.

Removing the contigs already accounted for by SSUs, plasmids, and viruses, we are left with 5,231 contigs. Notably, 4,888 of the total Kaiju eukaryotic contigs were classified by MMseqs2, but 178 were at no rank, 949 at root, and 2,526 at the superkingdom level. Taking into account the contigs that were already assigned, there were 3,223 contigs that were assigned as eukaryotic by Kaiju and ambiguous by MMseqs2, and 886 contigs assigned as eukaryotic by Kaiju and no assignment by MMseqs2 (subtracting those classified by geNomad as virus or plasmid), for a total of 4,109 eukaryotic contigs by Kaiju.

Manual curation + geNomad + MMseqs2 + Kaiju

- 40986 Bacteria
- 316 Archaea
- 4260 Eukaryota
- 369 Unassigned prokaryotic sequences (plasmids)
- 46475 Viruses (or 27103, conservatively)
- 15571 Unclassified

The Kaiju results add some ambiguity to the number of archeal and viral contigs. However, since the Kaiju assignments for archaea were within the same order of magnitude as the MMseqs2 results, we did not examine them closely. Kaiju assigns 23,499 contigs as viruses and 25,407 as unclassified, so it is tempting to say that many of these unclassified contigs are probably viral. We found that of the unclassified Kaiju contigs, 9,915 were assigned as viral by geNomad, and 8,458 overlapped with contigs that could not be classified by the other methods we used. Based on this information, we estimate that 33,414 contigs are viral, for a more conservative estimate than the geNomad results. This is closer to the previous conservative estimate of 27,103 viral contigs.

Combining everything, our final estimates for the taxonomy of the contigs are

- 38% Bacteria
- 0.3-0.7% Archaea
- 4% Eukaryota
- 25-43% Virus
- 14% Unclassified or ambiguous

References

1. Rasmussen, A. N. & Francis, C. A. Genome-Resolved Metagenomic Insights into Massive Seasonal Ammonia-Oxidizing Archaea Blooms in San Francisco Bay. *mSystems* **7**, e0127021 (2022).
2. Yarza, P. *et al.* Uniting the classification of cultured and uncultured bacteria and archaea using 16S rRNA gene sequences. *Nat. Rev. Microbiol.* **12**, 635–645 (2014).
3. Oren, A. & Garrity, G. M. Valid publication of the names of forty-two phyla of prokaryotes. *Int. J. Syst. Evol. Microbiol.* **71**, (2021).
4. Camacho, C. *et al.* BLAST+: architecture and applications. *BMC Bioinformatics* **10**, 1–9 (2009).
